# Floral Scents of Deceptive *Arum maculatum* L. (Araceae) Shape a Taxonomically Diverse Visitor Community of Lesser Dung Flies (Sphaeroceridae) That Affects Its Reproductive Success

**DOI:** 10.1002/ece3.74139

**Published:** 2026-08-13

**Authors:** Danae Laina, Eva Gfrerer‐Burgschwaiger, Jindřich Roháček, Sigrid Mulitzer, Marc Gibernau, Hans Peter Comes, Anja C. Hörger, Stefan Dötterl

**Affiliations:** ^1^ Department of Environment and Biodiversity University of Salzburg Salzburg Austria; ^2^ Department of Entomology Silesian Museum Opava Czech Republic; ^3^ Laboratory of Sciences for the Environment CNRS – University of Corsica Ajaccio France; ^4^ Centre for Climate Change Resilience (CC:R) University of Salzburg Salzburg Austria

**Keywords:** *Arum maculatum*, Diptera, floral scent, fly‐pollination, reproductive success, *Spelobia cambrica*, *Spelobia clunipes*, Sphaeroceridae

## Abstract

Most angiosperms are pollinated by animals, with insects being the most abundant and diverse pollinators. However, our knowledge about the diversity of Diptera as floral visitors and pollinators, and about their attraction to flowers, is still limited. Fly‐deceptive plant species, such as *Arum maculatum* , are ideal model systems to explore these interactions, as their floral scents often mimic (parts of) the ecological niches associated with the dipteran life cycle (e.g., mating, breeding). In this study, we investigated the species and sex identity of lesser dung flies (Sphaeroceridae), the second most abundant dipteran floral visitor family of *A. maculatum* after Psychodidae, across 10 populations of this plant species north and south of the Alps. Using previously published data on floral scent and fruit set, we also tested in a single southern *A. maculatum* population, where psychodids were limited and sphaerocerids abundant visitors, whether the abundance of the two predominant fly species, *Spelobia clunipes* and *S. cambrica* (and their sexes), correlates with inter‐individual differences in floral scent compounds and fruit set. Of the 20 sphaerocerid species identified across all populations, nine are newly reported as floral visitors of *A. maculatum* , and two represent new country‐specific records. The sex ratio of the flies was balanced within each region, and in the focal southern population the abundance of *S. clunipes* and *S. cambrica* correlated with the absolute amount of certain floral scent compounds, with some differences between the sexes of these species. The abundance of both sexes of only *S. clunipes* correlated with fruit set, suggesting that this species is a potential pollinator of *A. maculatum* . Overall, our data show that *A. maculatum* is visited by a high diversity of sphaerocerids both north and south of the Alps. This diversity is higher than for psychodid flies, the most abundant visitors of this plant species. Our data suggest that *A. maculatum* exhibits a mixed deceptive strategy.

## Graphical abstract

We identified lesser dung fly (Sphaeroceridae) visitors of the deceptive *Arum maculatum* and asked, whether the communities of these flies vary geographically, are shaped by floral scents, and predict the reproductive success of this plant species. Our data revealed that sphaerocerid visitors of *A. maculatum* are taxonomically highly diverse (20 species, 13 genera). We also identified floral scent compounds potentially involved in the attraction of the most abundant species, and showed that one of them correlated with the plants' reproductive success.

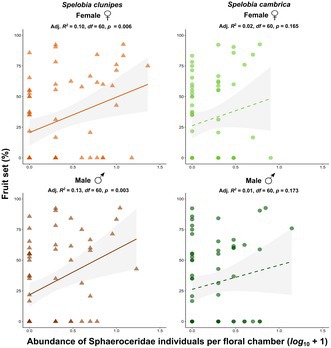

## 1 Introduction

The importance of animals in pollination and floral divergence is well‐documented, with up to 90% of flowering plants being animal‐pollinated (Ollerton et al. 2011; Schiestl and Johnson 2013; Tong et al. 2023). Insects comprise the majority of pollinators and have played a key role in floral evolution, with current estimates indicating that insect pollination has prevailed for *c*. 86% of total angiosperm evolutionary time (Peña‐Kairath et al. 2023; Stephens et al. 2023). Information on the diversity of pollinating insects has recently increased alongside the discovery of novel and uncommon pollinators, including, among others, bugs (Etl et al. 2022; Ishida et al. 2009), cockroaches (Xiong et al. 2020), and crickets (Micheneau et al. 2010). The vast majority of pollinating insects belong to four orders, namely Hymenoptera, Diptera, Coleoptera, and Lepidoptera, with an estimated total of *c*. 343,900 pollinating species (Ollerton 2017). Among these orders, Diptera with *c*. 150,000 species in *c*. 150 families (Pape et al. 2009) are thought to have the lowest diversity of visitor/pollinator species (*c*. 55,000) (Ollerton 2017). However, this likely represents a gross underestimate (Ollerton 2017; Ssymank et al. 2008; Wardhaugh 2015) due to three interconnected reasons. First, some dipteran families considered particularly important pollinators, such as Syrphidae (hoverflies) and Bombyliidae (bee flies), have received disproportionate attention in pollination studies; in consequence, secondary dipteran visitors are often neglected, together with data on their relative contribution to plant fitness and floral phenotypes (Kay and Anderson 2025; Larson et al. 2001; Orford et al. 2015). Second, when Diptera are not the primary pollinators, researchers frequently overlook their abundance, sex, and species identity (Orford et al. 2015; Raguso 2020; Wardhaugh 2015). And third, species identification in Diptera is challenging; hence, individuals are often only identified to family or genus level in pollination/ecological studies (Matallana‐Puerto et al. 2025; Meier et al. 2006; Pape et al. 2009; Rupp et al. 2021; Wardhaugh 2015).

In the last decade, new fly pollination systems (myiophily) mediated by olfactory cues have been described (for reviews see Dötterl and Gershenzon 2023; Raguso 2020). By identifying both the attractive floral scent compounds and the pollinating flies to species level, researchers have gained deeper insights into the ecology of floral scent as well as the biology and ecology of these insects (e.g., Inouye et al. 2015; Raguso 2020). This, in particular, applies to deceptive fly pollination systems, where flowers chemically imitate a reward related to the ecological habits of the flies, typically a food source, a brood site, or a mating signal (e.g., Jersáková et al. 2006, 2009; Jürgens et al. 2006, 2013; Urru et al. 2011). To deceive their fly pollinators, plants may mimic carrion and feces (e.g., for oviposition/food; Johnson and Jürgens 2010), fungi (e.g., for oviposition; Policha et al. 2016, 2019), fruits (e.g., for oviposition/food; reviewed in Goodrich and Jürgens 2018), sex pheromones (e.g., for mating, Martel et al. 2019; Matsumoto et al. 2023), aphid (alarm) pheromones (e.g., for oviposition/food; Stökl et al. 2011; Jiang et al. 2020), and injured insects, such as bugs (Oelschlägel et al. 2015), honeybees (Heiduk et al. 2016) and ants (Mochizuki 2025) (e.g., for food/egg maturation, kleptomyiophily/insect‐host mimicry; reviewed in Vrecko et al. 2025).

However, understanding and determining what type of resource a mimicked “reward” represents (e.g., food, oviposition, and/or mating) is challenging given the diverse diets and habitat/resource needs of Diptera depending on the stage of their life cycle (i.e., larval versus adult), sex identity, and/or physiological state (Brodie et al. 2016; Davis et al. 2023; Stökl et al. 2010; Vrecko et al. 2025). For example, *Aristolochia microstoma* (Aristolochiaceae) emits floral volatiles known from invertebrate carrion and mostly attracts female and male phorid flies (*Megaselia* spp., Phoridae), but it is unclear whether such a substrate serves these flies as a food source and/or oviposition site (Rupp et al. 2021). Moreover, the mimicked “reward” may cover a combination of substrates, as it has been demonstrated for *A. palaestinium*, whose yeast fermented‐like scent matches the food and oviposition preferences of several drosophilid species (Stökl et al. 2010). Despite recent insights (e.g., Matallana‐Puerto et al. 2025), our understanding of the broad spectrum of pollination and visitation by Diptera remains limited. This, in particular, applies to deceptive systems, where large gaps of knowledge persist regarding visitor identity, sex, and diversity, the specific role of floral scent in their attraction, and the extent to which these factors influence plant reproductive success (Ollerton 2017; Raguso 2020; Wardhaugh 2015).

In this study, we address these issues by investigating the interaction between *Arum maculatum* L. (Araceae) and a so far less considered group of its fly visitors/pollinators, namely lesser dung flies (Sphaeroceridae). The pollination system of this plant species is relatively well‐studied; it emits a highly diverse dung‐like scent (i.e., with *c*. 300 scent compounds recorded) to attract its fly pollinators (sapromyiophily), and exhibits specialized interactions with female Psychodidae, and particularly with *Psychoda phalaenoides* (Linnaeus, 1758), which is considered the main (effective) pollinator (Diaz and Kite 2002; Espíndola et al. 2011; Gibernau et al. 2004; Laina et al. 2022; Szenteczki et al. 2021). Given the high abundance and female‐biased ratios of visiting *P. phalaenoides* , i.e., flies that lack functional mouthparts and, therefore, cannot feed (Oosterbroek 2006), *Arum maculatum* is considered a brood‐site mimic, although egg‐laying behavior has never been observed in visiting flies (Chartier et al. 2013; Diaz and Kite 2002; Gibernau et al. 2004). It has been shown that species composition and abundances within visitor assemblages of *A. maculatum* are highly variable and involve the attraction of other dipteran families, such as Sphaeroceridae (Laina et al. 2022; Roháček et al. 1990; Ruiz 2009), especially in populations where the primary pollinators (i.e., Psychodidae) are less abundant (Laina et al. 2022). However, it remains unclear whether the mimicked scents signal a similar substrate to these flies and/or if their modes of attraction differ. In our previous study, comparing *A. maculatum* populations north versus south of the Alps, Sphaeroceridae were, overall, identified as the second most frequent dipteran visitors in the south after Psychodidae, whose abundance appeared to be low (Laina et al. 2022). The abundance of Sphaeroceridae even matched or exceeded that of psychodids in two southern populations (i.e., LIM and DAO, respectively) and correlated with fruit set across the southern region, suggesting that sphaerocerids are not only floral visitors but also pollinators of *A. maculatum* (Laina et al. 2022). In addition, differences in floral scent between northern and southern *A. maculatum* populations (Gfrerer et al. 2021) may reflect different selection regimes, likely driven by the higher diversity yet lower visitor abundance in the south versus north (see also Gross et al. 2016). Yet, detailed information is still lacking regarding the species identity and diversity of visiting sphaerocerids, the role of floral scent in the attraction of these taxa as well as the potential influence of single species on the plant's reproductive success.

Here, we determined the species identity (and, if possible, the sex) of all Sphaeroceridae individuals previously collected and recorded across 10 *A. maculatum* populations north and south of the Alps (Laina et al. 2022). Furthermore, we took advantage of existing floral scent and fruit set data from a southern (DAO) population (Gfrerer et al. 2021; Laina et al. 2022), where particularly high numbers of sphaerocerid visitors have been recorded, and where higher amounts of emitted scent attract more sphaerocerid individuals (Laina et al. 2022; Gfrerer et al. 2023). For this population, we investigated the extent to which the abundance and sex of the two predominant species correlate with individual plant differences in floral scent compounds and fruit set.

Overall, we tested several a priori predictions. First, in our previous study (Laina et al. 2022), we found that psychodid species compositions (i.e., relative abundances) differed between *A. maculatum* populations north versus south of the Alps. Hence, we hypothesized that sphaerocerid species compositions would likewise differ between these two regions, and may potentially also vary among populations within each region. Second, based on the high numbers of sphaerocerid species previously recorded as visitors in *A. maculatum* inflorescences (i.e., 16 species, Germany, Roháček et al. 1990; 19 species, Spain, Ruiz 2009), we expected a high sphaerocerid species diversity in *A. maculatum* populations north and south of the Alps. Third, we expected a female‐biased sex ratio, assuming that individuals of this family are deceived by brood‐site mimicry, as reported for the West Mediterranean endemic *A. pictum* (Quilichini et al. 2010). And finally, for the focal southern population (DAO), we predicted that the abundance of predominant flower‐visiting sphaerocerid species correlates (1) with the amount of floral scent compounds, especially those under phenotypic selection (Gfrerer et al. 2021) and/or eliciting physiological responses in the antennae of the sphaerocerid *Coproica ferruginata* (Stenhammar, 1855) (Gfrerer et al. 2022); and, at least in part, (2) with inter‐individual variation in fruit set.

## 2 Materials and Methods

### 2.1 Study Plant Species, Collection, and Identification of Sphaeroceridae

*Arum maculatum* is widely distributed across Europe, reaching eastward to northern Turkey and western Caucasus (Boyce 2006; POWO 2026). This perennial monocot, which flowers from April to May in Central Europe, has a deceptive pollination strategy that strongly depends on olfactory cues (Gfrerer et al. 2021; Kite et al. 1998). It has a dung‐like floral scent that lures mainly dipteran pollinators (Psychodidae, Sphaeroceridae) (sapromyiophily) to temporarily trap them, without offering a reward (Diaz and Kite 2002; Espíndola et al. 2011; Gfrerer et al. 2021; Laina et al. 2022). The trap‐like inflorescence is formed by a modified bract (“spathe”) surrounding a spike (“spadix”); the latter bears an apical “appendix”, followed downwards by male (sterile and fertile) and female (sterile and fertile) flowers (Gibernau et al. 2004; Lack and Diaz 1991; Marotz‐Clausen et al. 2024; Sowter 1949). On the first day of anthesis, the spathe opens and reveals the appendix, while the male and female flowers remain enclosed at its base, forming the floral chamber (FC) (Gibernau et al. 2004; Lack and Diaz 1991). During this time, the appendix produces heat (thermogenesis) and emits a dung‐like scent that attracts the floral visitors, which eventually slip and fall into the floral chamber where they potentially deposit pollen on the receptive female flowers (protogyny) (Bermadinger‐Stabentheiner and Stabentheiner 1995; Lack and Diaz 1991; Sowter 1949). In the chamber, the insects remain trapped until the following day, when the pollen is released (i.e., male stage) and dusted onto their bodies while the spathe and the sterile flowers wither, allowing them to escape. After successful pollination, fruits develop into red berries until summer (Lack and Diaz 1991; Sowter 1949).

In this study, we morphologically determined the species and sex identity of all Sphaeroceridae individuals (Papp 2008; Roháček 1983; Roháček and Marshall 1986) previously collected (Laina et al. 2022) during three flowering seasons (2017, 2018, and 2019; see details below) from the floral chambers (i.e., individuals) of each of five *A. maculatum* populations north versus south of the Alps [i.e., North: NEC, Horb am Neckar (sampled in 2018); BUR, Burg Hohenstein (2018); MUR, Murnau am Staffelsee (2018) (all Germany); JOS, Josefiau (2017, 2018, 2019; Austria); RUM, Rümikon (2018; Switzerland); South: DAO, Daone (2017, 2018); LIM, Limone in Piemonte (2017); BER, Santa Maria Hoè (2019); MON, Montese (2017); UDI, Udine (2018) (all Italy); see also Tables S1 and S2 in Laina et al. 2022]. Where sex identification was not possible, individuals were categorized as “unidentified sex”.

In the southern DAO population (45°57.60′ N, 10°34.80′ E), where Sphaeroceridae individuals were most numerous (Laina et al. 2022), we further analyzed the visitation of these flies to inflorescences with known information on both floral scent (for details see Gfrerer et al. 2021, 2023) and fruit set (percentage of female flowers that developed into fruits with seeds; Gfrerer et al. 2021; Laina et al. 2022). For these analyses, we only included inflorescences sampled in 2017 (*N*_FC_ = 62, with or without sphaerocerids; see specific sample sizes below), and fruit set refers to infructescences collected in the summer of this year.

### 2.2 Statistical Analyses

Differences in the composition of visiting sphaerocerid species between northern and southern *A. maculatum* populations were assessed using permutational analysis of variance (PERMANOVA, 9999 permutations) in PRIMER v. 6.0 (Clarke and Gorley 2006). In this analysis, a Bray–Curtis similarity matrix of the relative abundance of the sphaerocerid species (i.e., absolute abundance of each species divided by the total number of sphaerocerids in a floral chamber) served as input. The term *region* was used as a fixed grouping factor, and *population nested within region* was used as a random factor for stratification by population. Only populations with at least five floral chambers (FCs; i.e., plant individuals) containing sphaerocerids and only inflorescences that trapped at least one sphaerocerid individual were included in this analysis (i.e., North: JOS, *N*_FC_ = 8; MUR, *N*_FC_ = 10; South: DAO, *N*_FC_ = 55; LIM, *N*_FC_ = 12). For populations JOS and DAO, we only considered FCs sampled in 2017 to exclude potential temporal variation arising from the different years of sampling (see above and Herrera 1988; Price et al. 2005). To test for differences in the number of female and male sphaerocerids within each region, a Wilcoxon‐Mann–Whitney test (WMW; R package COIN, Hothorn et al. 2008) was performed separately for each region, including all sampled FCs in which sphaerocerids were recorded (North: *N*_FC_ = 28; South: *N*_FC_ = 81).

As most of the sphaerocerid individuals were collected from the southern DAO population (*N*_Sphaer_ = 489; Table S1), and most of them were identified as either *Spelobia clunipes* (Meigen 1830) or *S. cambrica* (Richards 1929) (Table S1), all subsequent statistical analyses were based on this population and these two sphaerocerid species. As above, only FCs sampled in 2017 were included (*N*_FC_ = 62). To visualize similarities and dissimilarities in sex composition of the two focal sphaerocerid species in the FCs, non‐metric multidimensional scaling (NMDS) in PRIMER v. 6 was employed. For this purpose, a pairwise Bray–Curtis similarity matrix was calculated, using the relative abundance of each sphaerocerid group (i.e., female and male *S. clunipes* ; female and male *S. cambrica*) per FC that trapped at least one individual of *S. clunipes* and/or *S. cambrica* (*N*_FC_ = 50). To assess whether the sexes of each species are similarly attracted to the FCs, we performed linear regressions in R v. 4.3.0, using log‐transformed abundances (log_10_ + 1) (R Core Team 2023).

To test whether there is a correlation between the absolute amount (peak area; TIC, total ion current) of a scent compound emitted and the abundance of each sphaerocerid group (number of inflorescences with both scent and visitor data available: *N*_FC_ = 61), a two‐step approach was employed, given that biases can arise due to the large number of variables (i.e., scent compounds) and potential correlations among them (i.e., multicollinearity) (Graham 2003). After calculating a Pearson correlation matrix, significant correlations (*p* < 0.05) were further evaluated using linear models. The abundance of each sphaerocerid group was treated as response variable and the absolute amount of a scent compound as explanatory variable. Only compounds that occurred in at least 10 floral chambers were included (*N* = 36, see also Section 3). Sphaerocerid abundances and scent emission were log‐transformed (log_10_ + 1) to reduce the skewness in the data set.

To assess whether females and/or males of each *Spelobia* species are attracted to inflorescences with particular scent profiles (*N*_FC_ = 61), we performed distance‐based linear models (Legendre and Andersson 1999; McArdle and Anderson 2001) in PRIMER (function “distLM”) with 9999 permutations. To this aim, we calculated a Bray–Curtis similarity matrix of the log‐transformed (log_10_ + 1) absolute amounts of the scent compounds and used the log‐transformed (log_10_ + 1) abundances of the four sphaerocerid groups as explanatory variables.

Finally, to test whether females and/or males of each *Spelobia* species affect the fruit set of *Arum maculatum* individuals, we performed linear models with the log‐transformed abundances of the four sphaerocerid groups as explanatory variables and fruit set as the response variable. Following Laina et al. (2022), fruit set was coded as zero if no infructescence was available.

## 3 Results

### 3.1 Sphaerocerid Abundance, Species, and Sex Identification in the Floral Chambers of *Arum maculatum*

**FIGURE 1:**
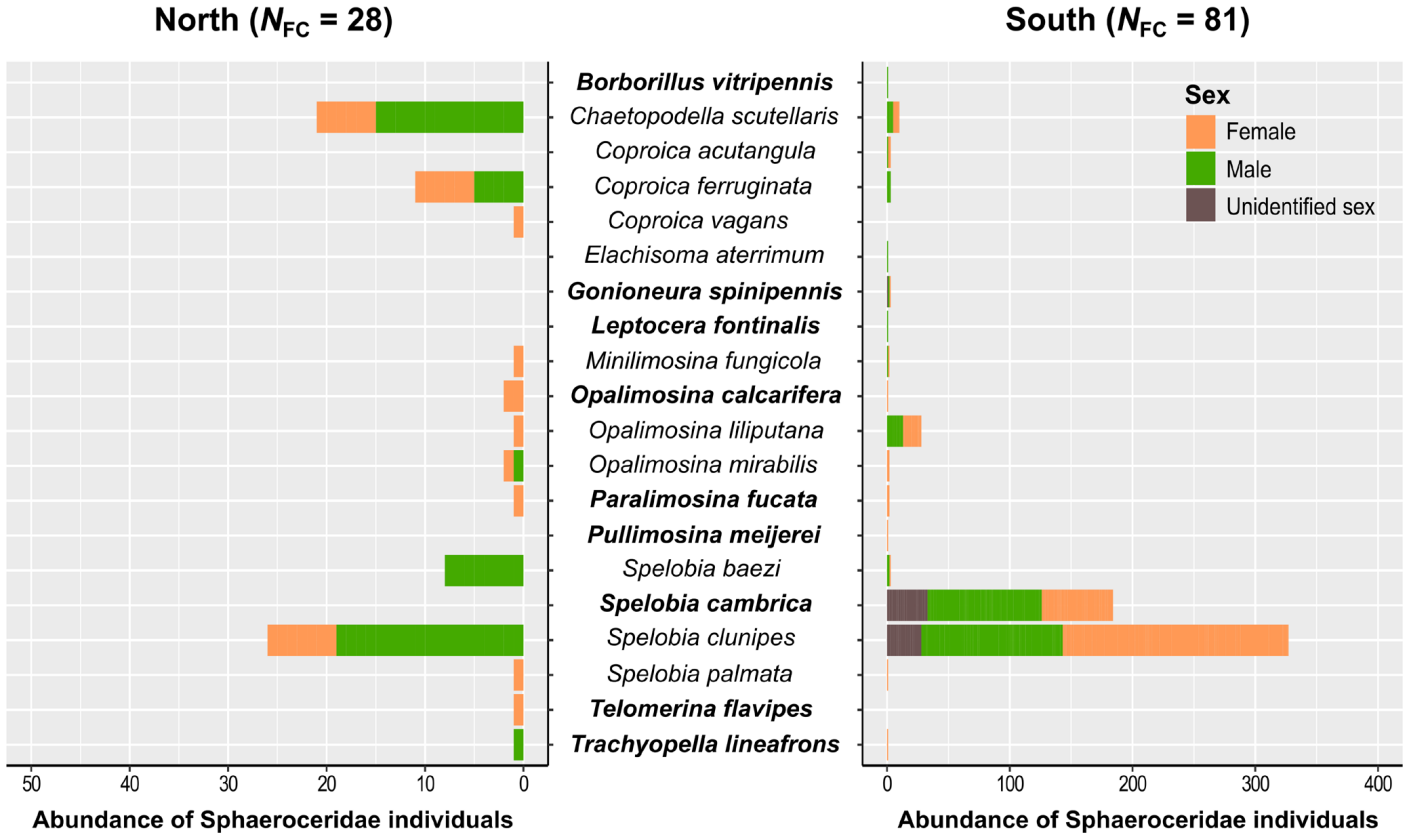
Species and sex abundance of Sphaeroceridae flies in floral chambers (FCs) of *Arum maculatum* populations north versus south of the Alps. Note the different scales of the *x*‐axes. Species in bold represent taxa newly identified as floral visitors of *A. maculatum* (see also Roháček et al. 1990; Ruiz 2009). Taxonomic authorships are provided in Table S1.

In total, 651 sphaerocerid individuals were collected from *Arum maculatum* floral chambers (FCs) north (*N*_FC_ = 28) and south (*N*_FC_ = 81) of the Alps (Figure 1, Table S1). There was no significant difference in the composition (relative abundances) of sphaerocerid species between the two regions (pseudo‐*F*_1_,_84_ = 0.89, *p* = 0.67); however, their relative abundances in the FCs differed significantly among populations within the regions (pseudo‐*F*_2_,_84_ = 15.38, *p* < 0.001). The number of female versus male individuals was similar in each region and not significantly different (North: *N*_Female_ = 28, *N*_Male_ = 49, WMW: *Z* = −0.74, *p* = 0.46; South: *N*_Female_ = 275, *N*_Male_ = 237, WMW: *Z* = 0.37, *p* = 0.71). Most sphaerocerid individuals (*N*_Sphaer_ = 574) originated from the five southern populations, with DAO having the largest representation (*N*_Sphaer_ = 489; *N*_FC_ = 60, with at least one sphaerocerid specimen recorded over two flowering seasons; Table S1), followed by LIM (*N*_Sphaer_ = 68; *N*_FC_ = 12) (Figure 1, Table S1). In the north, 77 sphaerocerid individuals were recorded across the five study populations, with most of them originating from MUR (*N*_Sphaer_ = 42; *N*_FC_ = 10), followed by JOS (*N*_Sphaer_ = 24; *N*_FC_ = 12) (Figure 1, Table S1).

All collected sphaerocerid specimens (*N*_Sphaer_ = 651) could be assigned to a total of 20 species, belonging to 13 genera (Figure 1, Table S1). Of these, 11 were recorded in both southern and northern regions, seven species only in the south, and two only in the north. In the south, the most common sphaerocerid species was *Spelobia clunipes* with a total of 327 individuals (*N*_Female_ = 184; *N*_Male_ = 115; *N*_Unidentified sex_ = 28), followed by *S. cambrica* (only recorded in DAO) with 184 individuals (*N*_Female_ = 58; *N*_Male_ = 93; *N*_Unidentified sex_ = 33), and *Opalimosina liliputana* with 28 individuals (*N*_Female_ = 15; *N*_Male_ = 13), while all other species were recorded with a maximum of 10 individuals. In the north, *Spelobia clunipes* (*N*_Total_ = 26; *N*_Female_ = 7, *N*_Male_ = 19) and *Chaetopodella scutellaris* (*N*_Total_ = 21; *N*_Female_ = 6, *N*_Male_ = 15) were the most abundant sphaerocerid species. *Coproica ferruginata* occurred with 11 individuals (*N*_Female_ = 6, *N*_Male_ = 5), *Spelobia baezi* with eight (only males), and all other species with a maximum of two individuals. Notably, in almost all species with at least four individuals recorded, both sexes were found, with the exception of *S. baezi* (see Figure 1, Table S1).

**FIGURE 2:**
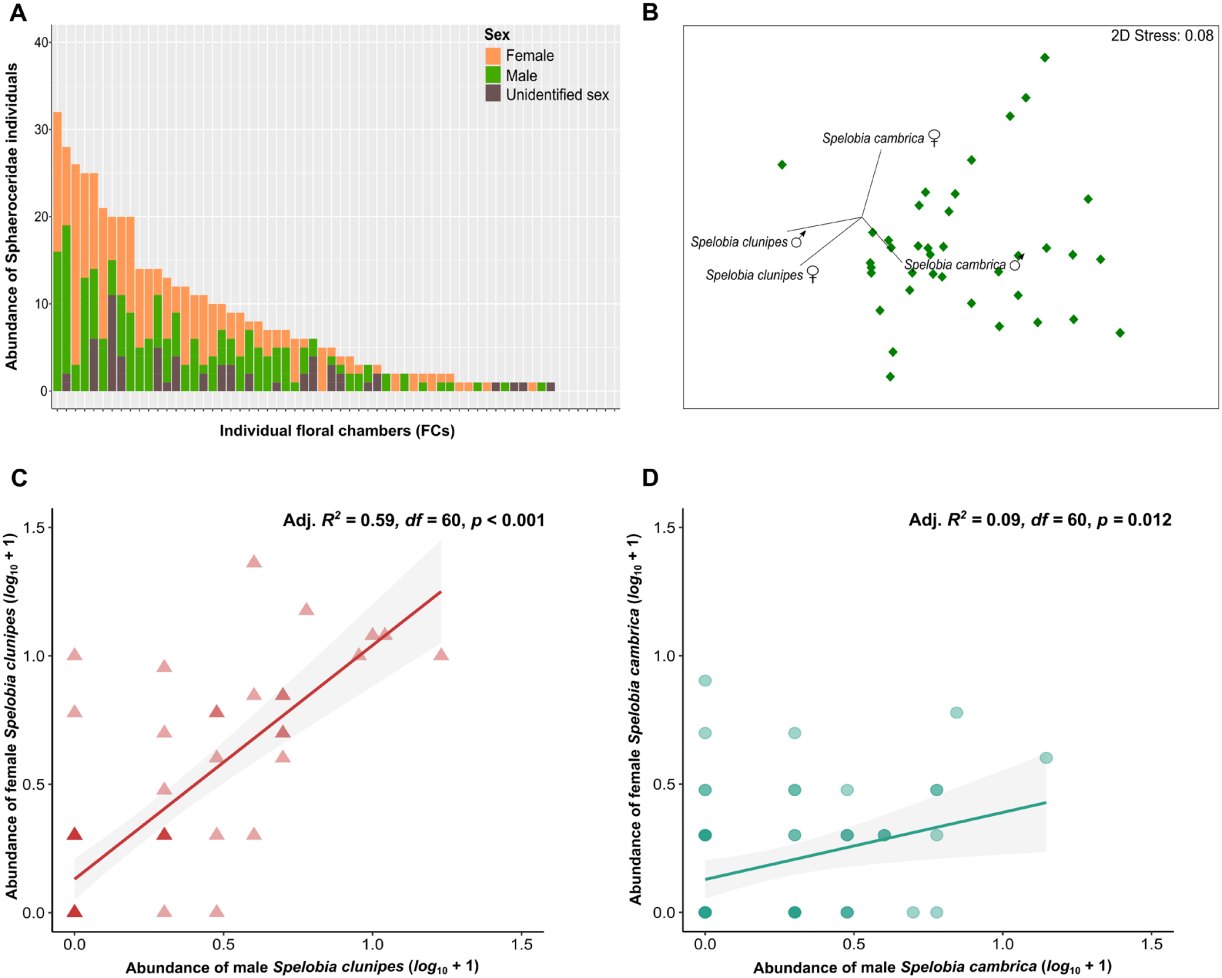
Sphaeroceridae visitation in the southern Daone (DAO) population of *Arum maculatum* (flowering season 2017). (A) Variations in the abundance and sex of Sphaeroceridae individuals collected from individual floral chambers (FCs), each represented by a single bar (*N*_FC_ = 62). (B) Non‐metric multidimensional scaling (NMDS; 2D stress = 0.08) visualizing similarities and dissimilarities in the relative abundance of female and male *Spelobia clunipes* , and female and male *S. cambrica*, in individual FCs (*N*_FC_ = 50; with at least one individual of either female/male *S. clunipes* or female/male *S. cambrica* recorded). Linear correlations between the absolute abundance of female and male *S. clunipes* (C), and female and male *S. cambrica* (D), in individual FCs (*N*_FC_ = 62). Darker triangles in (C) and darker circles in (D) represent overlapping data points. Shaded areas represent 95% confidence intervals (CIs). The adjusted *R* ^2^ (Adj. *R* ^2^) and the respective *p*‐value refer to the fit of the linear model and the latter also to the significance of the explanatory variable [i.e., abundance of male *S. clunipes* (C)/*S. cambrica* (D)].

In the following, we will focus on the DAO population (*N*_FC_ = 62 with or without sphaerocerids; flowering season 2017), where most sphaerocerid individuals and in particular the two most abundant species ( *S. clunipes* , *S. cambrica*) were recorded (Figure 2). Here, the absolute abundances of females and males correlated significantly in both species, but more strongly in *S. clunipes* (slope = 0.91, adj. *R* ^2^ = 0.59, df = 60, *p* < 0.001; Figure 2C) than in *S. cambrica* (slope = 0.26, adj. *R* ^2^ = 0.09, df = 60, *p* = 0.012; Figure 2D). Thus, inflorescences that attracted a high number of *S. clunipes* females often also attracted high numbers of conspecific males, although to a lesser extent in the case of *S. cambrica* (Figure 2B–D). Accordingly, in the ordination based on the relative abundances of the two sexes of these species in single inflorescences, females and males of *S. clunipes* had more similar loadings than those of *S. cambrica* (see vectors Figure 2B).

### 3.2 Effects of Floral Scent Compounds on Female/Male Abundance of *Spelobia* spp.

In the southern DAO population, the abundances of all four tested sphaerocerid groups (i.e., female and male *Spelobia clunipes* ; female and male *S. cambrica*) correlated with the absolute amount of a few single floral scent compounds. Specifically, of the 257 compounds recorded in DAO (Gfrerer et al. 2021), 36 showed significant correlations across the four groups (Table 1 and Table S2, Figures S1–S3). These scent compounds accounted for an average of 51% of the relative scent emission of inflorescences in this population (based on Gfrerer et al. 2021). Twenty‐two scent compounds were significantly correlated with the abundance of female *S. clunipes* . Of these, 21 showed positive correlations (i.e., the higher the emission of a given scent compound, the higher the abundance of female *S. clunipes* ), while one unknown compound (UNK1794) was negatively correlated (i.e., the higher the emission, the lower the abundance of female *S. clunipes* ) (Tables 1 and S2, Figure S1). The positively correlated compounds included an aliphatic alcohol (4‐methylhexan‐1‐ol), eight monoterpenoids (2,6‐dimethylocta‐2,6‐diene, 3,7‐dimethyloct‐2‐ene, *α*‐citronellene, *β*‐citronellene, *α*‐terpinene, *γ*‐terpinene, sabinene, and *β*‐phellandrene), one sesquiterpenoid (*β*‐caryophyllene), and one nitrogen‐bearing compound (indole), while the remaining 10 compounds were unknowns (Table 1 and S2, Figure S1).

**TABLE 1:** Significant linear regressions between the abundances of female and male *Spelobia clunipes* , and female and male *S. cambrica* and the absolute amount (log_10_ + 1) of single floral scent compounds emitted by *Arum maculatum* inflorescences (*N* = 61) in the southern Daone (DAO) population. *N*_Inflor_ refers to the number of inflorescences for which the respective scent compound was recorded. The adjusted *R* ^2^ (Adj. *R* ^2^) and the respective *p*‐value (in parentheses) refer to the fit of the linear model and the latter also to the significance of the explanatory variable (i.e., absolute amount of scent compound). Values in bold indicate negative correlations (see also Table S2). For all models, the residual standard error was calculated with 59 degrees of freedom.

Scent compounds	*N*_Inflor_	Female *S. clunipes*	Male *S. clunipes*	Female *S. cambrica*	Male *S. cambrica*
		Adj. *R* ^2^ (*p*)	Adj. *R* ^2^ (*p*)	Adj. *R* ^2^ (*p*)	Adj. *R* ^2^ (*p*)
*Aliphatic components*					
2‐Heptanol	52	—	—	0.080 (0.016)	—
4‐Methylhexan‐1‐ol	44	0.053 (0.041)	—	—	—
*Monoterpenoids*					
2,6‐Dimethylocta‐2,6‐diene	61	0.088 (0.012)	—	—	—
3,7‐Dimethyloct‐2‐ene	61	0.143 (0.002)	—	—	—
*α*‐Citronellene	59	0.080 (0.016)	—	—	—
*β*‐Citronellene	61	0.057 (0.035)	—	—	—
*β*‐Myrcene	20	—	—	—	0.056 (0.037)
*α*‐Terpinene	15	0.073 (0.020)	0.066 (0.026)	—	—
*γ*‐Terpinene	23	0.071 (0.021)	0.069 (0.023)	—	—
*α*‐Terpinolene	18	—	—	0.068 (0.024)	—
Citronellal	18	—	0.070 (0.022)	—	—
Sabinene	43	0.087 (0.012)	0.104 (0.007)	—	—
*β*‐Phellandrene	24	0.086 (0.012)	0.091 (0.010)	—	—
*Sesquiterpenoids*					
*β*‐Caryophyllene	60	0.052 (0.042)	—	—	—
*Nitrogen‐bearing compounds*					
Indole	61	0.087 (0.012)	0.051 (0.044)	—	—
*β*‐Lutidine	28	—	0.074 (0.019)	—	—
*Irregular terpene*					
Hexahydrofarnesylacetone	29	—	—	—	**0.050** (**0.047**)
*Aromatic components*					
*p*‐Cresol	44	—	0.058 (0.034)	—	—
*Unknown compounds*					
UNK1014	14	0.071 (0.021)	0.049 (0.047)	—	—
UNK1030	41	0.094 (0.009)	—	—	—
UNK1068	33	0.087 (0.012)	0.059 (0.033)	—	—
UNK1124	11	0.065 (0.026)	—	—	—
UNK1135	45	0.062 (0.029)	—	—	—
UNK1240	15	—	0.065 (0.027)	—	—
UNK1440	43	—	0.071 (0.021)	—	—
UNK1460	11	0.072 (0.020)	—	—	—
UNK1517	14	—	—	0.071 (0.022)	—
UNK1526	11	—	—	0.056 (0.038)	—
UNK1596	18	0.090 (0.011)	—	—	—
UNK1640	32	—	0.060 (0.031)	—	—
UNK1667	17	0.081 (0.015)	0.053 (0.041)	—	—
UNK1794	48	**0.076** (**0.018**)	—	—	—
UNK1854	29	—	—	—	0.062 (0.029)
UNK863	26	—	—	0.085 (0.013)	—
UNK960	23	0.053 (0.041)	—	—	—
Unknown53	59	0.056 (0.037)	—	—	—

The abundance of male *S. clunipes* correlated positively with a total of 14 scent compounds, eight of which were common in conspecific females (i.e., *α*‐terpinene, *γ*‐terpinene, sabinene, *β*‐phellandrene, indole, UNK1014, UNK1068, UNK1667; Tables 1 and S2, Figure S2). The remaining six included the monoterpenoid citronellal, the nitrogen‐bearing compound *β*‐lutidine, the aromatic compound *p*‐cresol, and the unknown compounds UNK1240, UNK1440, and UNK1640 (Tables 1 and S2, Figure S2).

The abundance of female *S. cambrica* correlated positively with the emission of five scent compounds, including the aliphatic alcohol 2‐heptanol, the monoterpenoid *α*‐terpinolene and the unknown compounds UNK1517, UNK1526, and UNK863 (Tables 1 and S2, Figure S3). The abundance of male *S. cambrica* correlated positively with the emitted amount of the monoterpenoid *β*‐myrcene and the unknown UNK1854, but correlated negatively with the emission of the irregular terpene hexahydrofarnesylacetone (Tables 1 and S2, Figure S3).

The abundance of female *S. clunipes* was the only significant predictor of overall floral scent composition (pseudo‐*F*_59_ = 2.71, *p* = 0.016), while the abundances of male *S. clunipes* and both sexes of *S. cambrica* had no significant effect (all *p* > 0.05).

### 3.3 Effects of Female/Male Abundance of *Spelobia* spp. on Fruit Set

**FIGURE 3:**
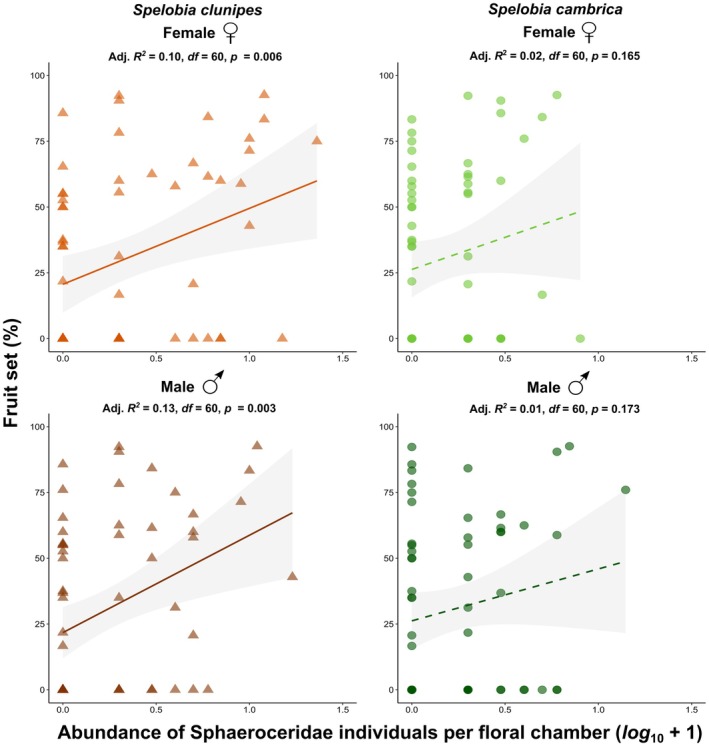
Linear regressions between fruit set (%) of *Arum maculatum* individuals in the southern Daone (DAO) population and the abundance (log_10_ + 1) of female *Spelobia clunipes* , male *S. clunipes* , female *S. cambrica,* and male *S. cambrica* recorded per respective floral chamber. Shaded areas represent 95% confidence intervals (CIs). The adjusted *R* ^2^ (Adj. *R* ^2^) and the respective *p*‐value refer to the fit of the linear model and the latter also to the significance of the explanatory variable [i.e., abundance of Sphaeroceridae individuals per floral chamber (log_10_ +1)].

Again, with focus on the DAO population, we found a highly significant positive correlation between differences in inter‐individual fruit set (*N* = 62) and the abundances of both sexes of *Spelobia clunipes* (female/male: slope = 28.88/37.01, adj. *R* ^2^ = 0.10/0.13, df = 60/60, *p* = 0.006/0.003). By contrast, no such significant relationship was observed for either sex of *S. cambrica* (female/male: slope = 24.44/19.74, adj. *R* ^2^ = 0.02/0.01, df = 60/60, *p* = 0.165/0.173) (Figure 3).

## 4 Discussion

In this study, a total of 20 Sphaeroceridae species were recorded as visitors of *Arum maculatum* in five populations from north and five populations from south of the Alps (Figure 1, Table S1). Eleven sphaerocerid species were recorded in both southern and northern regions, seven species only in the south and two only in the north, with higher abundances observed in the south (Figure 1, Table S1). The species composition of Sphaeroceridae differed significantly among populations within regions but, in contrast to our a priori prediction, no significant difference was detected between the two regions. In addition, unlike previously reported for Psychodidae (Espíndola et al. 2011; Laina et al. 2022), Sphaeroceridae showed no sex‐biased visitation ratios in either region. The southern Daone (DAO) population had the highest visitor abundance of Sphaeroceridae, dominated mainly by two species, namely *Spelobia clunipes* and *S. cambrica* (Figure 2). Overall, 22 floral scent compounds of *Arum maculatum* correlated significantly with the abundance of female *Spelobia clunipes* and 14 compounds with the abundance of male *S. clunipes* , while eight of them were common between females and males of this latter species (Tables 1 and S2; Figures S1 and S2). For *S. cambrica*, five and three significant correlations with scent compounds were detected for females and males, respectively (Tables 1 and S2; Figure S3). Interestingly, only female *S. clunipes* was found as a significant predictor of the floral scent composition. Furthermore, the abundances of both females and males of *S. clunipes* correlated significantly with fruit set, but no significant correlations were detected for either female or male *S. cambrica* (Figure 3).

### 4.1 Species and Sex Identification of Sphaeroceridae in *Arum maculatum*

Across our 10 study populations of *Arum maculatum* from north and south of the Alps, we recorded 20 visiting Sphaeroceridae species, in varying abundances, belonging to 13 genera, nine of which are here reported for the first time as floral visitors of this plant species (Roháček et al. 1990; Ruiz 2009; Figure 1, Table S1). The remaining 11 species (Figure 1) have previously been recorded as floral visitors of *A. maculatum* in South‐West Germany (Roháček et al. 1990) and/or in North‐East Spain (Ruiz 2009). Our findings reveal higher species diversity among visiting Sphaeroceridae, suggesting a more generalist floral attraction within this dipteran family compared to Psychodidae, for which only six visitor species in total were previously recorded in the same study populations (Laina et al. 2022).

Moreover, the absence of a sex‐biased visitor ratio for sphaerocerids (Figures 1 and 2; Table S1) differs from the known female‐biased visitation patterns of psychodids (Diaz and Kite 2002; Espíndola et al. 2011; Laina et al. 2022). This difference in the sex ratio of floral visitors between Sphaeroceridae and Psychodidae might indicate that the mode of attraction and, hence, the type of mimicry differs between the two dipteran families. That said, for adult individuals of *Psychoda*, feeding is highly improbable (Oosterbroek 2006). Consequently, it is possible that these flies visit sites chemically imitated by *Arum maculatum* only for oviposition and/or mating purposes. Instead, apart from oviposition (females only) and mating, female and male sphaerocerid individuals might be attracted by *A. maculatum* for feeding purposes as well, as both sexes of adults additionally feed on various decaying organic substrates, such as dung and fungi (e.g., Roháček et al. 2001; see also below), pointing to a mixed deceptive strategy (i.e., food/mating for male attraction, and food/mating/oviposition for female attraction). For sphaerocerids, the only other documented floral visitor sex ratios are reported for *A. pictum* , where a female‐biased ratio in *Coproica* spp. was attributed to floral scent‐mediated brood‐site mimicry or male repulsion (Quilichini et al. 2010); however, the latter is unlikely in our study system given the high number of males attracted by the plants.

Eleven of the 20 sphaerocerid species identified across the 10 study populations of *A. maculatum* were found in regions both north (Germany, Austria, Switzerland) and south (Italy) of the Alps (Figure 1). This is consistent with known presence records of most (i.e., nine) of these species in all four respective countries (i.e., *Chaetopodella scutellaris*, *Coproica ferruginata* , *Minilimosina fungicola* , *Opalimosina liliputana* , *O. mirabilis* , *Paralimosina fucata*, *Spelobia clunipes* , *S. palmata*, and *Trachyopella lineafrons* ) (Marshall et al. 2011; Roháček et al. 2001). However, our study provides new country‐specific records for *Opalimosina calcarifera* in Switzerland (RUM population) and for *Spelobia baezi* in Germany (MUR) (de Jong 2016; Gatt 2024; Marshall et al. 2011; Roháček et al. 2001; see also Figure 1, Table S1). It is unknown whether these new findings reflect recent colonization events or whether they have simply not been recorded until now (Marshall et al. 2011; Roháček et al. 2001; Roháček and Przhiboro 2022).

In this context it is also important to emphasize that our ‘region‐specific’ observations of the nine remaining spaerocerid species (i.e., north *or* south of the Alps; Figure 1, Table S1) cannot be explained by their overall distribution ranges, as these species are known to occur in both (northern *and* southern) regions of the studied *Arum maculatum* populations (Figure 1, Table S1; Marshall et al. 2011; Roháček et al. 2001). For example, although we recorded the abundant *Spelobia cambrica* only in the south (Italy), this species is also known from mountainous areas in Germany and Switzerland, and its occurrence can also be assumed for Austria (Bährmann 2016; Marshall et al. 2011; Roháček et al. 2001). As the life cycle of Sphaeroceridae is linked to soil (De Bruyn et al. 2001, 2002) and they are generally saprophagous (e.g., Roháček et al. 2001; see also above), our region‐specific observations of these species might rather be explained by certain characteristics of the local habitats, such as soil humidity, and the availability of suitable decaying substrates in the vicinity of the studied *Arum maculatum* populations (Buck 1997; De Bruyn et al. 2001; Roháček et al. 1990) or by temporal variation with respect to the stage of their life cycle during the sampling of the *A. maculatum* floral chambers (Buck 1997).

### 4.2 Effects of Floral Scent Compounds on the Abundance and Sex Ratios of *Spelobia* spp.

Of the 257 floral scent compounds previously reported for the southern Daone (DAO) population of *Arum maculatum* (Gfrerer et al. 2021), 36 correlated significantly with the abundances of the four tested sphaerocerid groups, i.e., female and male *Spelobia clunipes* , female and male *S. cambrica* (Tables 1 and S2; Figures S1–S3). Some of these compounds are known from potential feeding and oviposition/breeding substrates (e.g., carrion, decayed vegetation) of these two polysaprophagous fly species (Papp and Roháček 2021; Roháček 1983, 2011; Roháček and Przhiboro 2022), which they may therefore use to locate such substrates from a distance. For example, *α*‐ and *γ*‐terpinene, indole, and *p*‐cresol are known as scent components of herbivore dung (Dormont et al. 2004; Gfrerer et al. 2022; Jürgens et al. 2013), and 4‐methylhexan‐1‐ol, *α*‐terpinolene and/or *β*‐myrcene are (additionally) known from fungi (Breheret et al. 1997; Heddergott et al. 2014; Tables 1 and S2; Figures S1–S3). Interestingly, female *S. cambrica*, whose occurrence is related to montane forests and peat bogs, was previously recorded and collected from decayed fungi (Roháček 2011).

At DAO, strongly scented *Arum maculatum* individuals are known to attract more sphaerocerids (Gfrerer et al. 2023). However, which floral scent compounds can be perceived by different sphaerocerid species or sexes is largely unknown. The only evidence comes from a single female individual of *Coproica ferruginata* (a species here recorded mostly in the northern JOS population), in which a total of 36 scent compounds of *Arum maculatum* elicited antennal responses, and therefore, could be perceived by this fly individual (Gfrerer et al. 2022). Of these physiologically active scent compounds, 11 showed significant correlations across the four tested sphaerocerid groups (i.e., 2‐heptanol, *α*‐citronellene, *β*‐citronellene, 2,6‐dimethylocta‐2,6‐diene, 3,7‐dimethyloct‐2‐ene, *γ*‐terpinene, *β*‐caryophyllene, *p*‐cresol, indole as well as the unknowns UNK1030 and UNK1135; Tables 1 and S2; Figures S1–S3). Overall, this suggests that these compounds may also be perceived by individuals of female and/or male *Spelobia clunipes* or *S. cambrica*.

However, none of the correlating scent compounds were shared between *S. clunipes* and *S. cambrica* (Tables 1 and S2), as well as between the two sexes of *S. cambrica*. It is therefore feasible that different olfactory/substrate preferences exist, not only between the two species, but also between the females and males of *S. cambrica* (Tables 1 and S2; Figures S1–S3). In contrast, many of the correlations found in *S. clunipes* were shared between the sexes, pointing towards similar scent preferences (Tables 1 and S2; Figures S1 and S2). These results are also consistent with our finding that the abundances of females and males correlate more strongly in *S. clunipes* than in *S. cambrica* (Figure 2C,D). That said, in the present study, only changes in the abundance of female *S. clunipes* predicted variation in overall floral scent composition, while the latter had not previously been found as a predictor of the floral visitor composition in DAO (Gfrerer et al. 2023). However, it is unclear whether this result indicates a more suitable scent profile of *Arum maculatum* for attracting females of *Spelobia clunipes* (for which also the highest number of correlating compounds were detected), or whether the emission of specific scent compounds (or combinations thereof) is sufficient for the successful imitation of feeding, breeding and/or mating substrates, and thus for a somewhat more generalist attraction of sphaerocerid flies (Jürgens et al. 2013; Stökl et al. 2010; Urru et al. 2011).

For example, it was previously found that two scent compounds are under phenotypic selection in DAO, namely 4‐terpinenol and sabinene (Gfrerer et al. 2021). In fact, in the present study, the latter is among the significant correlating compounds for female and male *S. clunipes* . Considering that DAO is a population where the main pollinators (i.e., psychodids) are scarce (Laina et al. 2022), it is tempting to speculate that floral adaptations in *Arum maculatum* might evolve in response to less‐effective pollinators to maximize plant fitness, a phenomenon known as 'adaptive generalization' (Aigner 2001; Kay and Anderson 2025; Ohashi et al. 2021; Wenzell et al. 2024). While Gfrerer et al. (2022) demonstrated that the floral scent of *A. maculatum* differs significantly between DAO and the northern *Psychoda phalaenoides* ‐dominated JOS population, more research is needed to test this hypothesis, also regarding the aspect of temporal pollinator variation (Price et al. 2005; Szenteczki et al. 2021). Furthermore, determining whether olfactory preferences differ among the four tested sphaerocerid groups (i.e., female and male *Spelobia clunipes* ; female and male *S. cambrica*), requires electrophysiological analyses (i.e., gas chromatography‐electroantennographic detection, GC‐EAD), ideally coupled with the identification of behaviourally active compounds.

### 4.3 Effects of *Spelobia* spp. Abundance on Fruit Set

Our results indicate that differences in fruit set among *Arum maculatum* individuals in the DAO population can only be explained by variations in the abundance of female and male *Spelobia clunipes* , but not of female or male *S. cambrica* (Figure 3). This is notable because both species were abundant visitors of *Arum maculatum* in this southern population and, like all sphaerocerid visitors of this plant species, are morphologically and trophically similar (Roháček 1983). In a previous study (Laina et al. 2022), we found considerable variation in the pollen load of sphaerocerid individuals (range = 0–385 pollen grains; *N*_Sphaer_ = 9). Since we did not differentiate between species and sexes in this previous study, we cannot distinguish between species/sex effects and inter‐individual differences within species/sexes in pollen loads. In the present study, the statistical differences in the effect of *Spelobia clunipes* and *S. cambrica* on fruit set might be due to differences in the behavior of the two species in the floral chambers of *Arum maculatum* (see also Laina et al. 2022), potentially resulting in different pollen loads. Similarly, individuals of *Spelobia clunipes* may visit these floral chambers more frequently than *S. cambrica*, making them more consistent pollinators. Clearly, further pollination experiments in this system are required to test this hypothesis.

## 5 Conclusions

Our study assessed the species and sex identity of Sphaeroceridae in *Arum maculatum* populations north and south of the Alps. Previously, this dipteran family was reported as the second most abundant visitor group across both regions overall, and equally or more abundant in certain southern populations (Laina et al. 2022). Twenty sphaerocerid species, belonging to 13 genera, were identified, of which nine have been reported for the first time as floral visitors of this plant species, and for two we provide new country‐specific occurrence records. Our study highlights the high diversity of secondary dipteran visitors in a specialized brood‐site deceptive plant species, notably without a sex‐biased ratio. Overall, this points to a more generalist attraction of sphaerocerid flies and suggests a mixed deceptive strategy, combining oviposition‐site mimicry with food and/or mating site mimicry. This is also consistent with our findings from the focal southern population (DAO), where both sexes of the predominant sphaerocerid species, namely *Spelobia clunipes* and *S. cambrica*, were attracted, with their abundance correlating with the absolute amounts of certain scent compounds. However, only *S. clunipes* seemed to have an influence on fruit set, suggesting that it could be a potential pollinator of this plant species. To this end, future studies should assess potential differences in substrate preferences (e.g., feeding, breeding) between sexes of *S. clunipes* and *S. cambrica*, how they perceive the floral scent of *Arum maculatum* , and whether they are efficient pollinators of this plant species. In conclusion, our study shows that sphaerocerids, as secondary visitors of *A. maculatum* , likely contribute to pollination and are attracted by floral scents that possibly signal not only oviposition/mating sites to these flies (as for psychodids) but additionally an adult food source. It remains to be explored whether variations in floral phenotype in this species (e.g., in scent, color, morphology) evolved as an adaptive response to secondary pollinators, particularly in populations where primary pollinators are scarce.

## Author Contributions

**Danae Laina:** conceptualization (equal), formal analysis (lead), investigation (lead), visualization (lead), writing – original draft (lead), writing – review and editing (equal). **Eva Gfrerer‐Burgschwaiger:** investigation (equal), writing – review and editing (supporting). **Jindřich Roháček:** investigation (equal), writing – review and editing (supporting). **Sigrid Mulitzer:** formal analysis (supporting), investigation (supporting), writing – review and editing (supporting). **Marc Gibernau:** conceptualization (supporting), writing – review and editing (equal). **Hans Peter Comes:** conceptualization (equal), funding acquisition (equal), writing – review and editing (equal). **Anja C. Hörger:** conceptualization (equal), funding acquisition (equal), supervision (equal), writing – review and editing (equal). **Stefan Dötterl:** conceptualization (equal), funding acquisition (equal), supervision (equal), writing – original draft (supporting), writing – review and editing (equal).

## Funding

This research was funded in whole or in part by the Austrian Science Fund (FWF) (10.55776/P30175). The research of J. Roháček was financially supported by the Ministry of Culture of the Czech Republic by institutional financing of long‐term conceptual development of the research institution (the Silesian Museum, MK000100595). All sampling conducted complies with the current laws of the respective countries.

## Conflicts of Interest

The authors declare no conflicts of interest.

## Supporting information


**Table S1:** Absolute abundances of sphaerocerid species and their sex composition in the floral chambers of *Arum maculatum* populations north and south of the Alps. “Unidentified” refers to those individuals, for which a sex identification was not possible. “N” refers to the number of *A. maculatum* floral chambers per population, where sphaerocerid individuals (*N*_Sphaer_ = 651) were recorded, followed (/) by the total number of floral chambers sampled per population (see also Tables S1 and S2 in Laina et al. 2022). Populations JOS and DAO include floral chambers sampled during three (2017, 2018, and 2019) and two (2017 and 2018) flowering seasons, respectively (see also Tables S1 and S2 in Laina et al. 2022 for further details). All other populations include floral chambers sampled in one flowering season (see text for details).
**Table S2:** Significant linear correlations between the abundance of each sphaerocerid group (log_10_ + 1) and the absolute amount of a scent compound emitted by *Arum maculatum* individuals in the southern Daone (DAO) population (see also Figures S1–S3). *N*_Inflor_ refers to the number of inflorescences for which the respective scent compound was recorded. The adjusted *R* ^2^ (Adj. *R* ^2^) and the respective *p*‐value (in parentheses) refer to the fit of the linear model and the latter also to the significance of the explanatory variable (i.e., absolute amount of scent compound). The standard error (SE) of the slope estimates is shown in brackets.
**Figure S1:** Linear regressions between the abundance of female *Spelobia clunipes* in the floral chambers of *Arum maculatum* (DAO; *N* = 61) and the absolute amount of emitted scent compounds (log_10_ + 1). Only significant correlations are shown (*p* < 0.05; see also Tables 1 and S1). Note the difference in the y‐axis for the unknown compound UNK1794. Shaded areas depict the 95% confidence intervals (CIs).
**Figure S2:** Linear regressions between the abundance of male *Spelobia clunipes* in the floral chambers of *Arum maculatum* (DAO; *N* = 61) and the absolute amount of emitted scent compounds (log_10_ + 1). Only significant correlations are shown (*p* < 0.05; see also Tables 1 and S1). Shaded areas depict the 95% confidence intervals (CIs).
**Figure S3:** Linear regressions between the abundances of (A) female *Spelobia cambrica* and (B) male *S. cambrica* in the floral chambers of *Arum maculatum* (DAO; *N* = 61) and the absolute amount of emitted scent compounds (log_10_ + 1). Only significant correlations are shown (*p* < 0.05; see also Tables 1 and S1). Note the difference in the *y*‐axis for the compound hexahydrofarnesylacetone. Shaded areas depict the 95% confidence intervals (CIs).

## Data Availability

The full scent data were published by Gfrerer et al. (2021) and can be found in Dryad Digital Repository (https://doi.org/10.5061/dryad.pnvx0k6kn). The sphaerocerid data supporting the conclusions of this article are included in this article and its Supporting Information.
