# Supplementary material for: Floral Scents of Deceptive Arum maculatum L. (Araceae) Shape a Taxonomically Diverse Visitor Community of Lesser Dung Flies (Sphaeroceridae) That Affects Its Reproductive Success
Source: Ecol Evol. 2026 Aug 13;16(8):e74139. doi: 10.1002/ece3.74139 (PMC13473747; doi:10.1002/ece3.74139)
Supplement: Supplementary file 1 [file ECE3-16-e74139-s001.docx]

***Supporting Information***

**Floral scents of deceptive *Arum maculatum* L. (Araceae) shape a taxonomically diverse visitor community of lesser dung flies (Sphaeroceridae) that affects its reproductive success**

Danae Laina^1^, Eva Gfrerer-Burgschwaiger^1^, Jindřich Roháček^2^, Sigrid Mulitzer^1^, Marc Gibernau^3^, Hans Peter Comes^1^, Anja C. Hörger^1*^, Stefan Dötterl^1*^

^1^Department of Environment and Biodiversity, University of Salzburg, Salzburg, Austria

^2^Department of Entomology, Silesian Museum, Opava, Czech Republic

^3^Laboratory of Sciences for the Environment, CNRS – University of Corsica, Ajaccio, France

^*^Corresponding authors: Assoc. Prof. Anja C. Hörger, [anja.hoerger@plus.ac.at](mailto:anja.hoerger@plus.ac.at)

Prof. Stefan Dötterl, [stefan.doetterl@plus.ac.at](mailto:stefan.doetterl@plus.ac.at)

|  |  | **Northern populations^a^** | | | | |  | **Southern populations^b^** | | | | |
| --- | --- | --- | --- | --- | --- | --- | --- | --- | --- | --- | --- | --- |
|  |  | BUR | JOS | MUR | NEC | RUM |  | BER | DAO | LIM | MON | UDI |
|  |  | *N*=1/16 | *N*=12/71 | *N*=10/15 | *N*=3/14 | *N*=2/15 |  | *N*=3/16 | *N*=60/73 | *N*=12/15 | *N*=2/15 | *N*=4/15 |
| **Sphaerocerid species** | **Sex** |  |  |  |  |  |  |  |  |  |  |  |
| *Borborillus vitripennis* (Meigen, 1830) | female | - | - | - | - | - |  | - | - | - | - | - |
|  | male | - | - | - | - | - |  | - | 1 | - | - | - |
| *Chaetopodella scutellaris* (Haliday, 1836) | female | 1 | - | 5 | - | - |  | - | 1 | 4 | - | - |
|  | male | - | 1 | 14 | - | - |  | - | 2 | 3 | - | - |
| *Coproica acutangula* (Zetterstedt, 1847) | female | - | - | - | - | - |  | - | 2 | - | - | - |
|  | male | - | - | - | - | - |  | - | 1 | - | - | - |
| *Coproica ferruginata* (Stenhammar, 1855) | female | 1 | 5 | - | - | - |  | - | - | - | - | - |
|  | male | - | 4 | 1 | - | - |  | 1 | - | - | 1 | 1 |
| *Coproica vagans* (Haliday, 1833) | female | - | - | - | - | 1 |  | - | - | - | - | - |
|  | male | - | - | - | - | - |  | - | - | - | - | - |
| *Elachisoma aterrimum* (Haliday, 1833) | female | - | - | - | - | - |  | - | - | - | - | - |
|  | male | - | - | - | - | - |  | - | - | 1 | - | - |
| *Gonioneura spinipennis* (Haliday, 1836) | female | - | - | - | - | - |  | - | 1 | - | - | - |
|  | male | - | - | - | - | - |  | - | 1 | - | - | - |
|  | unidentified | - | - | - | - | - |  | - | 1 | - | - | - |
| *Leptocera fontinalis* (Fallén, 1826) | female | - | - | - | - | - |  | - | - | - | - | - |
|  | male | - | - | - | - | - |  | - | 1 | - | - | - |
| *Minilimosina fungicola* (Haliday, 1836) | female | - | - | - | 1 | - |  | - | - | 1 | - | - |
|  | male | - | - | - | - | - |  | - | - | 1 | - | - |
| *Opalimosina calcarifera* (Roháček, 1975) | female | - | - | 1 | - | 1 |  | - | - | 1 | - | - |
|  | male | - | - | - | - | - |  | - | - | - | - | - |
| *Opalimosina liliputana* (Rondani, 1880) | female | - | - | - | - | 1 |  | - | 1 | 14 | - | - |
|  | male | - | - | - | - | - |  | - | 1 | 12 | - | - |
| *Opalimosina mirabilis* (Collin, 1902) | female | - | 1 | - | - | - |  | - | 1 | 1 | - | - |
|  | male | - | 1 | - | - | - |  | - | - | - | - | - |

**Table S1.** Absolute abundances of sphaerocerid species and their sex composition in the floral chambers of *Arum maculatum* populations north and south of the Alps. “Unidentified” refers to those individuals, for which a sex identification was not possible. “*N*” refers to the number of *A. maculatum* floral chambers per population, where sphaerocerid individuals (*N*_Sphaer_ = 651) were recorded, followed (/) by the total number of floral chambers sampled per population (see also Supplementary Tables 1 and 2 in Laina et al., 2022). Populations JOS and DAO include floral chambers sampled during three (2017, 2018, and 2019) and two (2017 and 2018) flowering seasons, respectively (see also Supplementary Tables 1 and 2 in Laina et al., 2022 for further details). All other populations include floral chambers sampled in one flowering season (see text for details).

| (*cont.*) |  | **Northern populations^a^** | | | | |  | **Southern populations^b^** | | | | |
| --- | --- | --- | --- | --- | --- | --- | --- | --- | --- | --- | --- | --- |
|  |  | BUR | JOS | MUR | NEC | RUM |  | BER | DAO | LIM | MON | UDI |
| *Paralimosina fucata* (Rondani, 1880) | female | - | - | - | - | 1 |  | - | 2 | - | - | - |
|  | male | - | - | - | - | - |  | - | - | - | - | - |
| *Pullimosina meijerei* (Duda, 1918) | female | - | - | - | - | - |  | - | - | - | - | 1 |
|  | male | - | - | - | - | - |  | - | - | - | - | - |
| *Spelobia baezi* (Papp, 1977) | female | - | - | - | - | - |  | - | - | 1 | - | - |
|  | male | - | - | 8 | - | - |  | - | - | 1 | 1 | - |
| *Spelobia cambrica* (Richards, 1929) | female | - | - | - | - | - |  | - | 58 | - | - | - |
|  | male | - | - | - | - | - |  | - | 93 | - | - | - |
|  | unidentified | - | - | - | - | - |  | - | 33 | - | - | - |
| *Spelobia clunipes* (Meigen, 1830) | female | - | 5 | 2 | - | - |  | 4 | 162 | 16 | 1 | 1 |
|  | male | 1 | 6 | 10 | 1 | 1 |  | 5 | 99 | 11 | - | - |
|  | unidentified | - | - | - | - | - |  | - | 28 | - | - | - |
| *Spelobia palmata* (Richards, 1927) | female | - | 1 | - | - | - |  | - | - | - | - | 1 |
|  | male | - | - | - | - | - |  | - | - | - | - | - |
| *Telomerina flavipes* (Meigen, 1830) | female | - | - | - | 1 | - |  | - | - | - | - | - |
|  | male | - | - | - | - | - |  | - | - | - | - | - |
| *Trachyopella lineafrons* (Spuler, 1925) | female | - | - | - | - | - |  | - | - | 1 | - | - |
|  | male | - | - | 1 | - | - |  | - | - | - | - | - |
| **Total number of Sphaeroceridae individuals per population** | | **3** | **24** | **42** | **3** | **5** |  | **10** | **489** | **68** | **3** | **4** |

**^a^ Northern populations:** BUR, Burg Hohenstein, Germany; JOS, Josefiau, Austria; MUR, Murnau am Staffelsee, Germany; NEC, Horb am Neckar, Germany; RUM, Rümikon, Switzerland.

**^b^** **Southern populations** (Italy): BER, Santa Maria Hoè; DAO, Daone; LIM, Limone in Piemonte; MON, Montese; UDI, Udine.

**Table S2.** Significant linear correlations between the abundance of each sphaerocerid group (*log*_10_ +1) and the absolute amount of a scent compound emitted by *Arum maculatum* individuals in the southern Daone (DAO) population (see also Figures S1-S3). *N*_Inflor_ refers to the number of inflorescences for which the respective scent compound was recorded. The adjusted *R^2^* (Adj. *R^2^*) and the respective *p*-value (in parentheses) refer to the fit of the linear model and the latter also to the significance of the explanatory variable (*i.e.*, absolute amount of scent compound). The standard error (SE) of the slope estimates is shown in brackets.

| **Scent compounds** | ***N_Inflor._*** | **Female *Spelobia clunipes*** | | **Male *Spelobia clunipes*** | | **Female *Spelobia cambrica*** | | **Male *Spelobia cambrica*** | |
| --- | --- | --- | --- | --- | --- | --- | --- | --- | --- |
|  |  | **Slope (SE)** | **Adj. *R^2^* (*p*-value)** | **Slope (SE)** | **Adj. *R^2^* (*p*-value)** | **Slope (SE)** | **Adj. *R^2^* (*p*-value)** | **Slope (SE)** | **Adj. *R^2^* (*p*-value)** |
| **Aliphatic components** |  |  |  |  |  |  |  |  |  |
| 2-Heptanol | 52 | - | - | - | - | 0.40 (0.16) | 0.080 (0.016) | - | - |
| 4-Methylhexan-1-ol | 44 | 7.99 (3.83) | 0.053 (0.041) | - | - | - | - | - | - |
| **Monoterpenoids** |  |  |  |  |  |  |  |  |  |
| 2,6-Dimethylocta-2,6-diene | 61 | 0.66 (0.25) | 0.088 (0.012) | - | - | - | - | - | - |
| 3,7-Dimethyloct-2-ene | 61 | 0.51 (0.15) | 0.143 (0.002) | - | - | - | - | - | - |
| *α*-Citronellene | 59 | 0.94 (0.38) | 0.080 (0.016) | - | - | - | - | - | - |
| *β*-Citronellene | 61 | 0.36 (0.17) | 0.057 (0.035) | - | - | - | - | - | - |
| *β*-Myrcene | 20 | - | - | - | - | - | - | 0.60 (0.28) | 0.056 (0.037) |
| *α*-Terpinene | 15 | 4.81 (2.01) | 0.073 (0.020) | 3.93 (1.71) | 0.066 (0.026) | - | - | - | - |
| *γ*-Terpinene | 23 | 2.80 (1.18) | 0.071 (0.021) | 2.35 (1.01) | 0.069 (0.023) | - | - | - | - |
| *α*-Terpinolene | 18 | - | - | - | - | 2.06 (0.89) | 0.068 (0.024) | - | - |
| Citronellal | 18 | - | - | 19.29 (8.20) | 0.070 (0.022) | - | - | - | - |
| Sabinene | 43 | 0.78 (0.30) | 0.087 (0.012) | 0.71 (0.25) | 0.104 (0.007) | - | - | - | - |
| *β*-Phellandrene | 24 | 4.88 (1.89) | 0.086 (0.012) | 4.25 (1.60) | 0.091 (0.010) | - | - | - | - |
| **Sesquiterpenoids** |  |  |  |  |  |  |  |  |  |
| *β*-Caryophyllene | 60 | 0.43 (0.21) | 0.052 (0.042) | - | - | - | - | - | - |
| **Nitrogen-bearing compounds** |  |  |  |  |  |  |  |  |  |
| Indole | 61 | 0.33 (0.13) | 0.087 (0.012) | 0.23 (0.11) | 0.051 (0.044) | - | - | - | - |
| *β*-Lutidine | 28 | - | - | 2.45 (1.02) | 0.074 (0.019) | - | - | - | - |
| **Irregular terpene** |  |  |  |  |  |  |  |  |  |
| Hexahydrofarnesylacetone | 29 | - | - | - | - | - | - | -2.81 (1.38) | 0.050 (0.047) |
| **Aromatic components** |  |  |  |  |  |  |  |  |  |
| *p*-Cresol | 44 | - | - | 0.28 (0.13) | 0.058 (0.034) | - | - | - | - |
|  |  |  | |  | |  | |  | |

| ***(cont.)*** |  |  | |  | |  | |  | |
| --- | --- | --- | --- | --- | --- | --- | --- | --- | --- |
| **Scent compounds** | ***N_Inflor._*** | **Female *Spelobia clunipes*** | | **Male *Spelobia clunipes*** | | **Female *Spelobia cambrica*** | | **Male *Spelobia cambrica*** | |
|  |  | **Slope (SE)** | **Adj. *R^2^* (*p*-value)** | **Slope (SE)** | **Adj. *R^2^* (*p*-value)** | **Slope (SE)** | **Adj. *R^2^* (*p*-value)** | **Slope (SE)** | **Adj. *R^2^* (*p*-value)** |
| **Unknown compounds** |  |  |  |  |  |  |  |  |  |
| UNK1014 | 14 | 8.54 (3.61) | 0.071 (0.021) | 6.30 (3.10) | 0.049 (0.047) | - | - | - | - |
| UNK1030 | 41 | 1.57 (0.59) | 0.094 (0.009) | - | - | - | - | - | - |
| UNK1068 | 33 | 3.58 (1.38) | 0.087 (0.012) | 2.60 (1.19) | 0.059 (0.033) | - | - | - | - |
| UNK1124 | 11 | 22.25 (9.78) | 0.065 (0.026) | - | - | - | - | - | - |
| UNK1135 | 45 | 3.27 (1.46) | 0.062 (0.029) | - | - | - | - | - | - |
| UNK1240 | 15 | - | - | 15.45 (6.81) | 0.065 (0.027) | - | - | - | - |
| UNK1440 | 43 | - | - | 3.72 (1.57) | 0.071 (0.021) | - | - | - | - |
| UNK1460 | 11 | 47.38 (19.87) | 0.072 (0.020) | - | - | - | - | - | - |
| UNK1517 | 14 | - | - | - | - | 10.79 (4.57) | 0.071 (0.022) | - | - |
| UNK1526 | 11 | - | - | - | - | 12.96 (6.09) | 0.056 (0.038) | - | - |
| UNK1596 | 18 | 14.03 (5.33) | 0.090 (0.011) | - | - | - | - | - | - |
| UNK1640 | 32 | - | - | 10.14 (4.60) | 0.060 (0.031) | - | - | - | - |
| UNK1667 | 17 | 9.96 (3.98) | 0.081 (0.015) | 7.18 (3.43) | 0.053 (0.041) | - | - | - | - |
| UNK1794 | 48 | -7.80 (3.21) | 0.076 (0.018) | - | - | - | - | - | - |
| UNK1854 | 29 | - | - | - | - | - | - | 3.90 (1.75) | 0.062 (0.029) |
| UNK863 | 26 | - | - | - | - | 2.63 (1.03) | 0.085 (0.013) | - | - |
| UNK960 | 23 | 5.75 (2.75) | 0.053 (0.041) | - | - | - | - | - | - |
| unknown53 | 59 | 0.45 (0.21) | 0.056 (0.037) | - | - | - | - | - | - |

**Figure S1.** Linear regressions between the abundance of female *Spelobia clunipes* in the floral chambers of *Arum maculatum* (DAO; *N* = 61) and the absolute amount of emitted scent compounds (*log*_10_ + 1). Only significant correlations are shown (*p* < 0.05; see also **Tables 1 and S1**). Note the difference in the y-axis for the unknown compound UNK1794. Shaded areas depict the 95% confidence intervals (CIs).

**Figure S2.** Linear regressions between the abundance of male *Spelobia clunipes* in the floral chambers of *Arum maculatum* (DAO; *N* = 61) and the absolute amount of emitted scent compounds (*log*_10_ + 1). Only significant correlations are shown (*p* < 0.05; see also **Tables 1 and S1**). Shaded areas depict the 95% confidence intervals (CIs).

**Figure S3.** Linear regressions between the abundances of **A.** female *Spelobia cambrica* and **B.** male *S. cambrica* in the floral chambers of *Arum maculatum* (DAO; *N* = 61) and the absolute amount of emitted scent compounds (*log*_10_ + 1). Only significant correlations are shown (*p* < 0.05; see also **Tables 1 and S1**). Note the difference in the y-axis for the compound hexahydrofarnesylacetone. Shaded areas depict the 95% confidence intervals (CIs).

**References**

Collin, J. E. (1902). Four new species of Diptera (Fam. Borboridae) found in Britain. *The Entomologist’s Monthly Magazine* **38**: 55-61.

Duda, O. (1918) *Revision der europäischen Arten der Gattung Limosina* *Macquart*. Vol. 10. Abhandlungen Zoologischen Botanischen Gesellschaft, Wien, Austria.

Fallén, C. F. (1826). *Supplementum Dipterorum Sveciae. Consentiente Ampl. Fac. Phil. Lund*. In Lyceo Carolino die XIII Dec. MDCCCXXVI. [Part. II.], 9-16 pp., Berlingiana, Londini Gothorum [= Lund].

Haliday, A. H. (1833). Catalogue of Diptera occuring about Holywood in Downshire.
*Entomological Magazine* **1**: 147-180.

Haliday, A. H. (1836). British species of the Dipterous tribe Sphaeroceridae. *Entomological Magazine* **3**: 315-336.

Laina, D., Gfrerer, E., Scheurecker, V., Fuchs, R., Schleifer, M., Zittra, C., Wagner, R., Gibernau, M., Comes, H. P., Hörger, A. C., Dötterl, S. (2022). Local insect availability partly explains geographical differences in floral visitor assemblages of *Arum maculatum* L. (Araceae). *Frontiers in Plant Science* **13**: 838391. doi:10.3389/fpls.2022.838391.

Meigen, J. W. (1830). *Systematische Beschreibung der bekannten europäischen zweiflügeligen Insekten.* Vol. 6. Schulz-Wundermann, Hamm, Germany.

Papp, L. (1977). Sphaeroceridae (Diptera) from Canary Islands. *Folia Entomologica Hungarica* **30**: 123-127.

Richards, O. W. (1927). New species of *Limosina* (Diptera) allied to *L. crassimana* Hal. *The Entomologist’s Monthly Magazine* **63**: 34-37.

Richards, O. W. (1929). Systematic notes on the Borboridae (Diptera) with description of new species of *Leptocera* (Limosina). *The Entomologist’s Monthly Magazine* **65**: 171-176.

Roháček, J. (1975). Three new species of the genus *Limosina* Macq. (Sphaeroceridae, Diptera) from Czechoslovakia. *Scripta Facultatis Scientiarum Naturalium Universitatis J. E. Purky- nianae Brunensis,* Biologia 2 **5**: 115-126.

Roháček J. (1983). A monograph and re-classification of the previous genus *Limosina* Macquart (Diptera, Sphaeroceridae) of Europe. Part II. *Beiträge zur Entomologie* **33**: 3–195.

Rondani, C. (1880). Species italicae ordinis dipterorum (*Muscaria* Rndn.) collectae et observatae. Stirps XXV. Copromyzinae Zett. *Bullettino della Società Entomologica Italiana* **12**: 3-45.

Spuler, A. (1925). Studies in North American Borboridae (Diptera). *The Canadian Entomologist* **57**: 99-104, 116-124.

Stenhammar, C. (1855). Skandinaviens Copromyzine granskade och beskrifne. *Kongliga Vetens- kaps-Akademiens Handlingar, Stockholm (ser. 3)* **1853**: 257-442.

Zetterstedt, J. W. (1847). *Diptera Scandinaviae. Disposita et descripta*. Vol. 6. 2163-2580 pp. Officina Lundbergiana, Lundae [= Lund].
